# Project and Prototype of Mobile Application for Monitoring the Global COVID-19 Epidemiological Situation

**DOI:** 10.3390/ijerph19031416

**Published:** 2022-01-27

**Authors:** Bartosz Sawik, Julia Płonka

**Affiliations:** 1 Department of Business Informatics and Engineering Management, AGH University of Science and Technology, 30-059 Krakow, Poland; 2 Department of Statistics, Computer Science and Mathematics, Public University of Navarre, 31006 Pamplona, Spain; 3 Haas School of Business, University of California at Berkeley, Berkeley, CA 94720, USA

**Keywords:** mobile application, data visualization, prototype, COVID-19

## Abstract

The purpose of this research is to analyze currently available solutions that help to monitor the global epidemiological situation, including travel restrictions, as well as proposing a new solution dedicated to users who want to keep updated with the current restrictions and COVID-19-related statistics. The analysis of existing tools is prepared from the perspective of practical usability for the end user. This paper consists of an overview of the tools and techniques of data visualization and demonstrates how to integrate them with practical business usage in a mobile application.

## 1. Introduction

In times when information is of significant value, the importance of data should be acknowledged. People have a preference for well-ordered and organized sets of data, as well as neatly presented charts and visualizations in order to better understand the world around us.

We live in an extraordinary time, which may have been predicted to some extent, but the current situation was generally unexpected. At the beginning of 2020, all of our lives changed—for some of us, dramatically—and we are living a different life now than we were this time two years ago. The COVID-19 pandemic exploded, and day by day it forced people to adjust their way of living to the new rules. Hospitals were required to adjust their tools and resources to the new reality as well, devising new approaches to deal with the rapidly changing situation [1,2]. Many new rules were introduced based on the core principles of social distancing, handwashing as often as possible, and wearing a face mask. However, these are not the only rules that people have had to follow. Each country has its own set of rules that are prepared based on medical research and expert knowledge. At the time when the virus first appeared, almost all of the countries in the world closed their borders completely or partially. The first country to isolate themselves from the world was North Korea, which, on 22 January 2020, decided not to let people in or out of the border in order to minimize the spread of the virus [3]. We all followed the news, watching how the lines on the graphs continued to increase over time and how the restrictions changed on a daily basis. A significant amount of research was conducted to evaluate how the epidemiological risk affected people’s behavior [4,5]. After some time, it began to become more difficult to remain up to date with the overload of information. All of the information that the politicians, TV presenters, and news websites were providing seemed to be very chaotic; there were no unified datasets with structured data that could be understood by everyone. At the same time, we were bombarded by new restrictions and rules introduced by our governments, and eventually it began to be more difficult to keep up to date with the guidance.

A number of solutions were presented that related to tracking the pandemic and path of the disease, including utilizing drones and special sensors that collect data from the areas of interest in real time [6]. Many existing solutions are based on geographical information systems, which are systems that create, manage, and analyze all types of data and connect them to a map, integrating locations with descriptive information. One such solution was developed in 2014 and focused on gathering and monitoring epidemiological surveys [4,7]. Based on what we already know about such systems, it would be advantageous to use this approach for tracking changes in restrictions, as well as COVID-19 statistics.

The coronavirus outbreak in 2020 was not the first time that epidemic risk was discussed and studied. It has been analyzed for a number of years, for example, how to detect emergency situations and epidemic threats and alert people of them [8], or summarizing country-specific epidemiological maps and measures for the control of tropical diseases [9]. Other associated problems that might cause epidemic outbreaks have also been analyzed and discussed, such as the quality of drinking water [10]. It was expected that we would have to face another significant pandemic at some point. Are we ever going to return to the “normal” we experienced prior to 2020? It is possible that the current restrictions will remain in place for an extended period of time. The situation is very dynamic, and it is difficult to predict what will happen within the next few years, even for the experts [11,12,13].

## 2. Materials and Methods

The authors consulted with epidemiologists and experts in medical sciences from the Poznan University of Medical Sciences in Poland, and UC San Diego School of Medicine in the USA, at the early stage of this research regarding the most important aspects that must be taken into account in order to create a useful prototype of a mobile application for monitoring the global COVID-19 epidemiological situation.

A well-prepared prototype may enable a reduction of the risks related to users’ acceptance and requirements in the information system development process. It might also help prevent unnecessary costs and generate constructive feedback. There are currently many techniques and tools that allow designers and developers to prepare a prototype of an application.

One of the first methods developed to prepare prototypes was prototyping on paper (POP). This approach involves drawing shapes representing the screen of a chosen device (mobile phone, tablet, laptop) on which the designers apply layers drawn on paper that imitate buttons and other parts of the user interface. POP does not require any electronic tools, as only paper, pens, and scissors are needed. Therefore, it is the simplest method of prototyping and has been used for many years. It is also possible to download free or paid templates online that support POP.

Besides prototyping on paper, there are many types of dedicated software for prototyping information system interfaces and mobile applications. Some of them simply allow the drawing of screens, buttons, and other elements of the user interface, and some also enable imitation of the transition between particular screens or steps of a process. These tools are used by user interface (UI) and user experience (UX) design teams on a daily basis.

The tool that is considered in this paper for the development of a prototype of the proposed application for monitoring the global epidemiological situation is Figma [14].

Figma [14] is an online tool that can also be used as a desktop application. In order to use it, the user needs to have a valid account. There are three options to choose from:

- Starter—the only free-to-use option, targeted to individuals who want to work on small projects. This is option used by the authors;
- Professional—allows an unlimited number of editors to work on the projects, with a license paid per user per month;
- Organizational—allows an unlimited number of teams to work on multiple projects.

Figma [14] is a tool that supports both UI and UX design, enables visualizations of the flows in the prototyped solutions, and allows the selection of different types of devices.

### 2.1. Survey on Feelings about Travel Restrictions Related to COVID-19

Research on people’s feelings and experiences of traveling during the pandemic was conducted in September 2021 by the authors of this paper using Google Forms. The link to the survey was shared via the authors’ Facebook account among friends, family, and groups for travelers. The request to fill in the questionnaire was targeted to people over the age of 18. Each of the respondents answered questions anonymously and independently. The first two questions of the survey were general in nature and unconnected to the field of traveling or mobile applications. The authors distinguished three age groups: 18–26 years old, which is the regular age for students in Poland; 27–35; and over 35. The second general question was about the respondents’ occupation, where there were four possible answers: student, employee, both student and employee, and unemployed. After the general questions, the definition of traveling was presented and explained, as there may have been some misunderstandings regarding this term. Following that, there were two questions about traveling before the pandemic, including frequency and main purpose. After that, there were four questions about traveling during the pandemic, also including queries about the restrictions. The terms “before the pandemic” and “during the pandemic” were properly explained, and the border point was set to March 2020, when the first traveling restrictions were introduced. The last part of the survey consisted of four questions concerning the source of information about the restrictions and the participants’ level of understanding of this information. It also included questions about the respondents’ expectations about the restrictions and the usage of mobile applications that were developed after the pandemic outbreak.

Before the coronavirus outbreak, 67% of the respondents traveled one or more times per year. The main purpose of the vast majority of these journeys was leisure and entertainment.

Assuming that “before the pandemic” was before March 2020, one and a half years passed from then to the moment of closing the survey. With that in mind, it might seem surprising that the vast majority of the respondents traveled once (20%) or more than once (67%) in that time. Only 12.5% did not travel during the pandemic. The main purpose of travel before and during the pandemic remained the same or similar.

#### 2.1.1. Restrictions while Traveling during the Pandemic

One of the last parts of the survey concerned the restrictions that the respondents encountered while traveling during the pandemic. The most common restrictions in the places to which the respondents traveled were covering their mouth and nose in public spaces, filling in a location form upon arrival, limiting the opening times of public premises, and curfew at the destination.

The vast majority of the respondents refrained from traveling because of compulsory quarantine upon arrival, no matter how long this lasted. A total of 35% of the respondents chose not to go on holiday to places where they would need to present a negative test result or where there was a curfew.

#### 2.1.2. Source of Information about the Restrictions

Approximately 90% of the respondents obtained information about the restrictions in place at their destination from news websites (bbc.com, onet.pl, cnn.com, worldometers.info, etc.). Almost 30% also used mobile applications as a source of information. Some respondents admitted to simply consulting their friends. Most of the respondents found that they understood the information about restrictions from the sources they were using well or very well (80%).

When asked whether they thought that the restrictions would remain in place for the next year or longer, almost all of the respondents answered ‘yes’ or ‘do not know for sure’. There was only one respondent who answered that the restrictions would be removed within the next year.

#### 2.1.3. Usage of the Most Popular COVID-Related Mobile Applications

The last question of the survey concerned mobile applications that are commonly available and are related to COVID-19. These applications were chosen for analysis in detail later in this paper. ‘Kwarantanna Domowa’ (Home Quarantine) happened to be the most popular amongst the respondents, which might be due to the fact that this tool is compulsory for everyone staying in quarantine. However, only 15% of the respondents reported using the tool. What is surprising is that only 6% of the respondents used STOP COVID-ProteGO Safe [15], despite this application being the most popular after Home Quarantine. The vast majority of the respondents did not report using any COVID-related mobile application.

Based on the survey results, it can be stated that the vast majority of the respondents believed that travel restrictions will remain in some form for a long time. People typically look for information about restrictions from sources such as news websites, but also obtain information from their friends and family. Most of the respondents did not use any COVID-19-related mobile application. In the next section, the authors analyze the mentioned tools to determine whether the low level of interest is due to the functionalities of the applications.

### 2.2. Comparison of COVID-19 Related Mobile Applications on the Market

#### 2.2.1. STOP COVID-ProteGO Safe

One of the most popular COVID-19-related mobile applications in Poland is STOP COVID-ProteGO Safe [15], introduced by the Ministry of Digital Affairs. This tool has had over one million downloads from Google Play Store, with almost 19,000 reviews (4.4/5 stars). In the Apple AppStore, it has been reviewed by over 6000 users (4.5/5 stars). It is the official Polish application that notifies users about their exposure to the coronavirus. It is intended for use in Poland. According to the publisher, it allows the smartphone to remember who the user met, inform users about their contact with infected persons, and advise what to do in such a situation. The application description states that the tool is safe and anonymous and does not allow the following actions:

- Collection of users’ data;
- Sharing of information with third parties;
- Tracking of users’ location;
- Accessing files or information kept on the device.

The application’s main functionality is tracking people that the user meets and notifying users about the potential risk of infection, based on Bluetooth technology. Once installed on the user’s smartphone (Bluetooth must be enabled), ProteGO Safe [15] searches the user’s surroundings for other phones that also have the app installed or are using any of its European equivalents. If it finds any such devices within 2 m, and if the user’s contact with the other individual lasts for at least 15 min, then this person will be saved in the application for the next 14 days. The data are stored in an encrypted manner. If the smartphone owner with an active ProteGO Safe [15] tests positive with coronavirus and shares this information in the application, other users who have had contact with that person will be notified and warned of the risk of infection.

Another functionality of ProteGO Safe [15] is the possibility of obtaining a referral for a COVID-19 test. It is prepared based on a short survey submitted by the user. It includes questions like whether the user has had contact with someone who has tested positive for coronavirus, or whether they have any symptoms or chronic ailments. If the answer to the questions is “yes”, then a referral will be generated, but the user needs to bear in mind that this may require them to self-isolate.

It is also possible to see daily reports in the application with the number of vaccinations (first and second dose, adverse reactions), cases, deaths, recoveries, and tests performed. The information can be retrieved for the whole country or the voivodeships and districts. ProteGO Safe [15] only shows statistics for Poland. The application also allows its users to store their health information in the form of a diary. They can enter such details as body temperature or symptoms in the scale from none to severe (runny nose, cough, shivering, or aching muscles). There is also a space for notes, for example, who the user has met recently or where they have been.

There is a section with important information, which includes recommendations on how to react when the user is feeling unwell (advice, phone numbers, etc.). It is also explained in detail how the application works and what the current restrictions are. ProteGO Safe [15] signposts the user for more specific information on travel restrictions and COVID-19 questions and answers to the Polish government website.

It is possible to set the application language to one of the following languages: German, English, Polish, Russian, Turkish, or Ukrainian. Some of the other applications that use Bluetooth technology to track the contact between its users are TraceTogether, CovidWatch, and Aarogya Setu.

#### 2.2.2. Home Quarantine

Another mobile application published by the Polish government is Home Quarantine—Poland. This one is also on the list of the most popular COVID-19-related applications, but not because of its interesting functionalities, but because it is a tool to validate people who are in quarantine. It was downloaded many times from Google Play Store with over 19,000 reviews (1.6/5 stars). In the Apple AppStore, it was reviewed by almost 2500 users (1.3/5 stars).

The application is intended only for people who, for some reason, are in quarantine. According to the current Polish law (since 1 April 2020), downloading and using the application in order to prove the user is following the quarantine requirements is mandatory for everyone who stays in Poland due to suspected SARS-CoV-2 infection. People who do not have a device that would allow them to use the application or do not have a mobile device at all may be exempt from this obligation.

Home Quarantine requires access to the user’s location and camera. After first launching the application, it informs the user about the requirements for using it and asks the user to grant a number of required permissions, such as location, multimedia, and camera, that are necessary to use the tool. After that, the application will ask the user to enter their phone number and 4-digit verification code sent by SMS. Once the user is verified and has confirmed that they should be in quarantine, the tasks to perform are shown on the main screen. The intention of such tasks is to confirm that the user is following quarantine rules. Home Quarantine sends multiple requests to complete a task each day. The user needs to take a picture of themselves and upload it to the application within 20 min of the first notification. The location of the mobile device is read by the application at the moment of uploading the picture.

Another feature of the Home Quarantine mobile application is that users can raise a request. It is possible for people who are in quarantine to report the need for contact with a psychologist or social worker, or the need for grocery shopping or a meal. The food options can only be used by people who do not have any other option, for example, living alone and have no one available to help them. The requests to contact a psychologist for help are forwarded to the local social welfare center.

Although the application is only intended for use in Poland, it is possible to choose a language from the list: English, Polish, Ukrainian, Russian, or Chinese.

#### 2.2.3. HealthLynked COVID-19 Tracker

Amongst the COVID-19-related mobile applications, there is another that seems to stand out for its popularity. HealthLynked COVID-19 Tracker is a mobile application that is available only in the Apple AppStore. It has been reviewed by almost 40,000 users (4.5/5 stars). It is a tool published by HealthLynked, an American web portal launched in 2016 in Naples, Florida. According to the website, their mission is “to improve healthcare by the transfer of accurate medical information between patients and their healthcare providers, improving medical practice efficiency, increasing access to quality healthcare, and facilitating accurate medical diagnosis”.

The application does not require logging in, but it might be necessary to use some of its functions. The main screen in the tool shows the basic statistics on coronavirus in the chosen country and across the world in general (cases, recoveries, deaths). There is also information about asymptomatic, symptomatic, and self-reported positive cases, but there are no data on that—the display shows 0. The data are updated every 30 min (according to the information in the application). Apart from their own internal statistics team in local areas around the world, the data are retrieved from sources such as:

- The CDC;
- Local state departments of health;
- Worldometer;
- Johns Hopkins Hospital;
- DXY DX Doctor;
- The WHO.

In the application, it is also possible to see statistics on the map; however, it is not very user friendly. When viewing the world map, the countries’ borders are not visible under the gigantic red spots, which represent the scale of volume and statistics in specific areas. Furthermore, when zooming into a smaller territory, especially in Europe, the spots are not accurate when it comes to the location of the countries they are representing.

Another feature that HealthLynked COVID-19 Tracker offers is a section with articles. This option is available after clicking the upper left button and then going to “COVID-19 Information”. There are seven publications by Michael Dent, M.D., who has worked in healthcare for over 30 years. Apart from general topics covering what the SARS-CoV-2 virus and COVID-19 disease are, the articles mention pregnancy and COVID-related information, the impact of the virus on pets, and general patient information.

In the application, there is also a “News” section, which gathers stories from news websites from around the world, such as The Times of India, New York Times, and Japan Today. The articles seem to be posted in the application on a simple condition—they have to be related to COVID-19. It is not possible to narrow the news feed by the location.

One of the outstanding features that is offered by the application is “Feeds”, where users can add posts and other users can like, comment, or share them. In order to be able to add posts on “Feeds”, one needs to be logged in. However, if someone does not want to enter their credentials and log in, it is still possible to interact with other users by accessing “Chat”. There are eight default groups split into topics, including Anxiety and Stress Release, Children and COVID-19, Home School, and Pregnancy. Under the name of the group, the number of active users currently in the chat are shown.

## 3. Results

### 3.1. Data Sources

#### 3.1.1. Existing Data Source—Statistics in Poland and Worldwide

In the case of COVID-19-related data in Poland, there were many speculations when it came to correctness of the data. Michal Rogalski [16], a recent high school graduate, is passionate about data and statistics. After the pandemic outbreak, he started collecting the numbers that were published daily by the Polish Ministry of Health. Starting from April 2020, he saved data from all of the Polish districts from various sources. He noticed some misstatements in the government publications, but these were usually small errors that were corrected and explained within a few days. However, in November 2020, it was noticed that roughly 22,000 cases were not included in the government publications. Because of this mistake, district sanitary stations stopped publishing detailed data. Despite the obstacles, Michal Rogalski [16] continued to collect the data, and today he runs a civic project in which he presents and updates various sets of COVID-19-related data in a publicly open file available in Google Drive. He also publishes analyses along with visualizations and short descriptions on his Twitter profile. A link to the mentioned database is also published on Twitter. The sources of information used to create the database are data based on the reports published by the Polish Ministry of Health, WSSE (Provincial Sanitary and Epidemiological Station) data, PSSE (District Sanitary and Epidemiological Station) data, provincial offices, and data collected after public information access requests.

The database currently (as of September 2021) consists of several sheets, including “Increase”, “Actual situation in Poland”, “Tests”, and the same for provinces and districts in Poland. It includes numbers of cases, deaths, daily increase, recoveries, and number of cases per 100,000 citizens.

**Figure 1:**
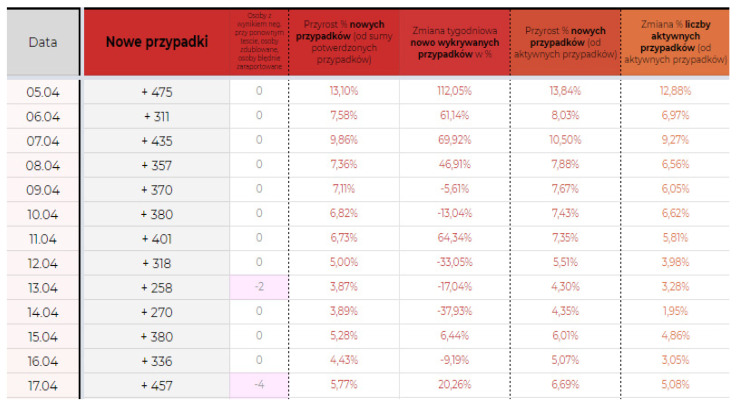
Example screenshot from the dataset with statistics from Poland, showing new cases, percentage of the increase of cases, weekly change of newly recognized cases, percentage increase of new cases compared to the active cases, and percentage change of number of active cases [16].

Figure 1 has presented an example of screenshot from the dataset with statistics from Poland, showing new cases, percentage of the increase of cases, weekly change of newly recognized cases, percentage increase of new cases compared to the active cases, and percentage change of number of active cases [16]. Explanation of columns in Figure 1 is the following: Day/Month; New Cases; Negative Result after Second Test; Percentage of the Increase of Cases; Weekly Change of Newly Recognized Cases; Percentage; Increase of New Cases Compared to the Active Cases; Percentage Change of Number of Active Cases.

The above dataset shows detailed statistics for Poland. Apart from this data source, the one that includes data from all around the world can also be used: https://ourworldindata.org/coronavirus-source-data (accessed on 24 January 2022) [17]. This data source is maintained by Our World in Data, a project of the Global Change Data Lab, a non-profit organization based in the United Kingdom. The organization gathers data on many critical issues that the world is facing—poverty, disease, hunger, climate change, war, existential risks, and inequality. The goal of the organization is to make the knowledge on such problems accessible and understandable. One of the issues about which the organization gathers data is the COVID-19 pandemic. Not only does it provide visualizations of the data, but also shares the raw data. The data can be downloaded and used from the GitHub repository: https://github.com/owid/covid-19-data/tree/master/public/data (accessed on 24 January 2022) [18]. It is updated daily and the sources of information are, inter alia:

- COVID-19 Data Repository by the Center for Systems Science and Engineering (CSSE) at Johns Hopkins University;
- European Centre for Disease Prevention;
- Official government reports.

These data sources can be used as an alternative to the first one by Michał Rogalski [16], and in addition, to present statistics from countries other than Poland.

#### 3.1.2. Proposed Data Source—Travel Restrictions

Apart from data sources that contain information about the statistics of the pandemic in Poland or in other countries, the authors propose a manually updated table that presents current travel restrictions. This would be the main feature that will distinguish the proposed mobile application from the existing ones. In order to be able to use such a table in any analytical tool such as Power BI or Tableau, it needs to be properly built and prepared.

The data sources for this information are official government online publications. Due to the dynamic changes in the epidemiological situation worldwide, the data will be updated every day (high dynamics), every two to three days (medium dynamics), or every week. Because of the non-standardized format of data, the table needs to be updated manually. In order to update the data, one needs to go through all of the government web pages from each country in the list. The list of countries includes all European Union countries, and additionally Switzerland, the United Kingdom, Ukraine, Russia, the United States of America, and Australia. The pages [19,20,21,22,23,24,25,26,27,28,29,30,31,32,33,34,35,36,37,38,39,40,41,42,43,44,45,46,47,48,49] to be checked for the data update.

The listed websites [19,20,21,22,23,24,25,26,27,28,29,30,31,32,33,34,35,36,37,38,39,40,41,42,43,44,45,46,47,48,49] are the first sources of information for the dataset. If there is no information provided or a website for a country is down, a European web page will also be checked: https://europa.eu/youreurope/citizens/travel/travel-and-covid/index_en.htm [50] (accessed on 24 January 2022).

It provides the option to choose a country of interest and check the answer to one of the following questions:

- What are the rules to enter this country with or without the EU Digital COVID certificate from an EU Member State or Schengen-associated country?
- As a third-country national coming from outside the EU and Norway, Switzerland, Iceland, and Liechtenstein, may I enter this country without exceptional restrictions?
- What are the rules if I go abroad from this country, and when I return from abroad?
- Can I transit through this country?
- May I enter this country by road, rail, air, or ship?

All of the information that this website shows is provided by the relevant departments of the European Commission and complemented by content provided by the authorities in every country it covers.

**Figure 2:**
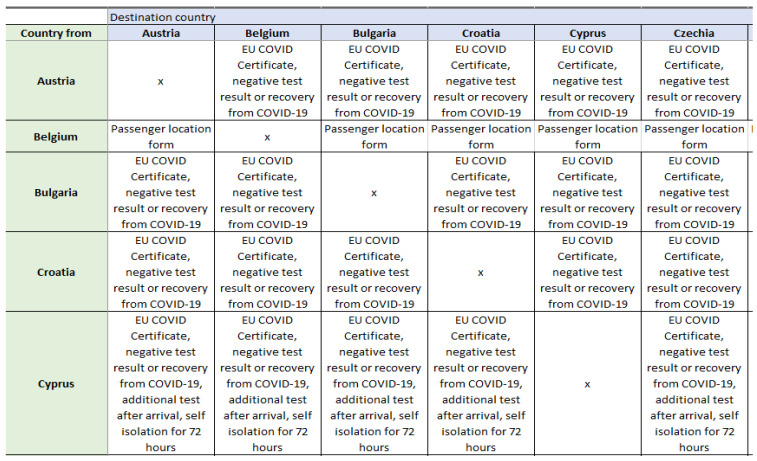
Example screenshot from the proposed dataset with restrictions in countries. Source: Authors own elaboration.

The dataset needs to be properly built, or at least structured, in order to be later used in one of the data visualizations tools. As this is a dataset that must contain information about travel restrictions to and from a chosen country, it can be presented as a matrix, where the columns represent countries “from” and rows represent countries “to”, i.e., countries are simultaneously both objects and metadata of those objects. To achieve this kind of structure, the columns have the same names as the rows, and the metadata for entries with the same name (diagonal of the matrix) will always be empty. The proposed dataset is built in an Excel file, where it can be easily and manually updated. An example of the prepared file is presented below. Figure 2 shows an example screenshot from the proposed dataset with restrictions in countries.

### 3.2. Data Visualization

Data visualization is a graphical representation of data (usually numeric). Its purpose is to explain, in an intelligible way, the information behind the data, that may lead to the drawing of conclusions. Data visualization is a very important area of data analysis because it allows the reader to quickly absorb the information, increases understanding of the presented data, and helps in the decision-making process. These are just a few of the many advantages of sufficient and effective data visualization.

According to Alexandru Telea, the visualization process is about “producing one or several images that should be able to convey insight into the considered process” [51]. In his book *Data visualization: Principles and practice*, the author distinguishes the following types of questions targeted by the visualization process:

- specific questions:
  ○ quantitative—what is the data’s minimum/average/distribution?
  ○ qualitative—do the data provide answers to a problem?
- discovering the unknown—what is in this dataset?

In the case of COVID-19-related datasets, the questions that are usually targeted are quantitative questions, e.g., what is the current number of infected people in the specific region? How many vaccinations have there been so far?

#### 3.2.1. Data Visualization Tools

**Figure 3:**
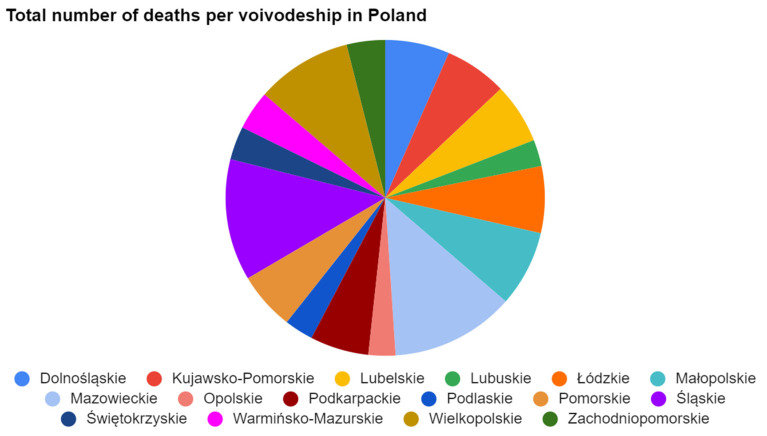
Example of a graphical visualization of data in Microsoft Excel, showing how fatal cases in Poland are distributed in particular voivodeships [15].

A data visualization tool is software whose purpose is to enable its user to visualize data and draw conclusions from it. A good visualization tool should join the functions of various steps of the data analysis or business intelligence processes, such as importing the data source, transforming the data and preparing them, visualizing the data using various graphical means, and even sometimes diving deep into the data using artificial intelligence. There are many data visualization tools on the market, but not all of them allow their users to complete the same actions. One of the most popular tools that enables graphical means of presenting data is Microsoft Excel. Although this software is not dedicated to data visualization, it is possible not only to manipulate the data and present them in tabular form, but also to create visual representations of them. This tool can be used to prepare charts and graphs, and even apply them on a map, but it is not the main purpose of this software and the visualization functionalities might be limited, for example, data sources that can be used must be included in the spreadsheet. To use the Microsoft Excel software, the user needs to have a valid MS Office license. An example of a chart illustrating COVID-19-related data is presented below. Figure 3 has presented an example of a graphical visualization of data in Microsoft Excel, showing how fatal cases in Poland are distributed in particular voivodeships.

Another popular tool for data visualization is Tableau. It was founded by the American software company Tableau Software in 2003. Unlike Microsoft Excel, Tableau is a tool that is dedicated to data analysis and visualizations. It is frequently used because of its relative simplicity and variety of possibilities. This software can be integrated with hundreds of data sources, for example:

- files in formats such as .xlsx, .xls, .csv, .xml;
- Microsoft SQL Server;
- Oracle database;
- Salesforce.com.

Many others are also included, such as PDF files. Tableau allows its users to create dashboards and reports with multiple pages. It is also possible to publish them, and the privacy level can be set so that everyone can see the content of the reports, even people without a Tableau account. It is possible to use Tableau for free, but with very limited features. To fully utilize this tool’s potential, the user needs to buy a license. Tableau is very often used by both private and corporate users. It works very well with big data sources and can also store the information using the Tableau server.

The next most popular data visualization tool is another solution introduced by Microsoft in 2011—Microsoft Power BI. The “BI” in the name stands for business intelligence. This tool is a business analytics service that aims to provide interactive visualizations and business intelligence capabilities. The assumption is that the interface of dashboards and reports would be simple enough for the end users, so that they can create their own analysis. Power BI is a part of the Microsoft Power Platform, along with Power Apps, Power Automate, and Power Virtual Agents. The tool allows its users to connect to multiple data sources, such as Microsoft SQL Server, R scripts, Python scripts, and many others. Because of its simplicity, characteristic of all MS Office tools, Power BI is available for use even by inexperienced users. Compared to Tableau, the interface might seem easier to learn. Its functionalities focus on data visualization, as the tool offers thousands of visualization types—besides those built into the desktop application, it is also possible to search for others online.

#### 3.2.2. Data Visualization Using Power BI

Because of the authors’ work experience and background, Microsoft Power BI was used to prepare and present an example visualization of the COVID-19-related data, which will be used later in the prototype of the mobile application for monitoring the epidemiological situation and travel restrictions around the world.

The first step in creating a dashboard in Power BI is to connect the pbix file to the data sources. The data sources were defined and presented in Section 2.1, and the same files were connected to the pbix file:

- Data from Poland by Michal Rogalski [16]—sheets used: Wzrost, Testy;
- Data from Our World in Data—named “Countries”, including information per country and date;
- Self-prepared dataset with the information about restrictions, named “Travel restrictions”.

**Figure 4:**
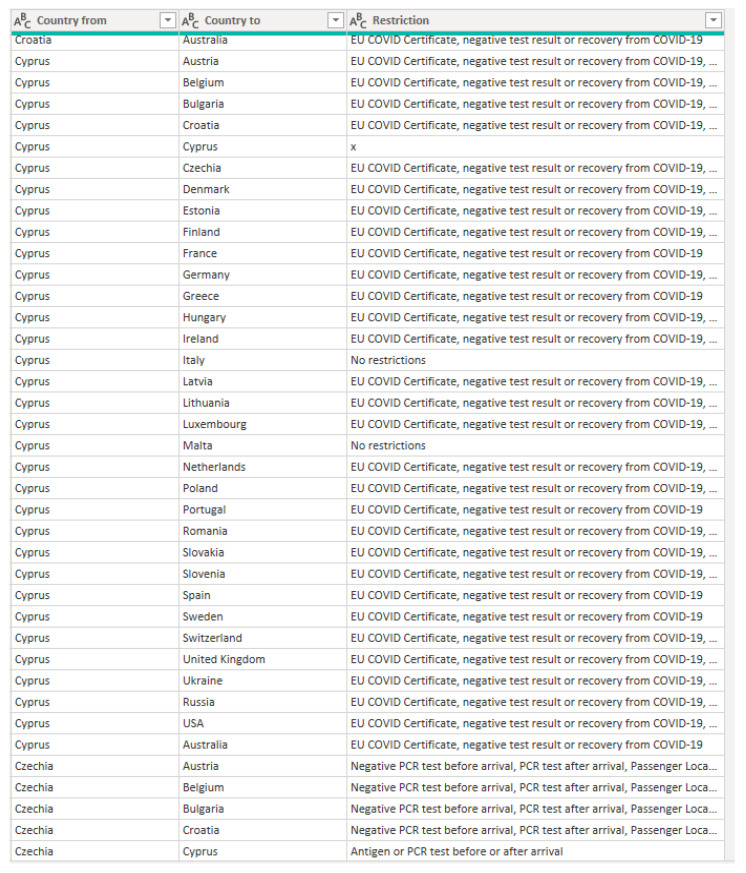
Part of the transformed table from the Power BI view. Source: Authors own elaboration.

After the connection is established, the transformation of the data is completed. In Power BI, it is possible to prepare the dataset using the Power Query editor. This allows the transformation of the data using either the M language or a wide range of built-in functions. In order to be able to correctly visualize information about restrictions from and to particular countries, the current form of the table is not sufficient. Therefore, a wide table, with columns as attributes of rows (column one—country “from”, the rest of the columns—countries “to”), needed to be transformed into a long table, with the result as in Figure 4. This figure presents part of the transformed table from the Power BI view.

Next, the other necessary actions were performed, such as setting the first rows as column headers and setting the correct data type. Two additional tables were added:

- Countries—list of countries as an export of the distinct values from the World table, column location;
- Calendar—a separate table with the start date set to 1 January 2020 (because no previous dates are included in either data sources), and the end date set with the function today).

**Figure 5:**
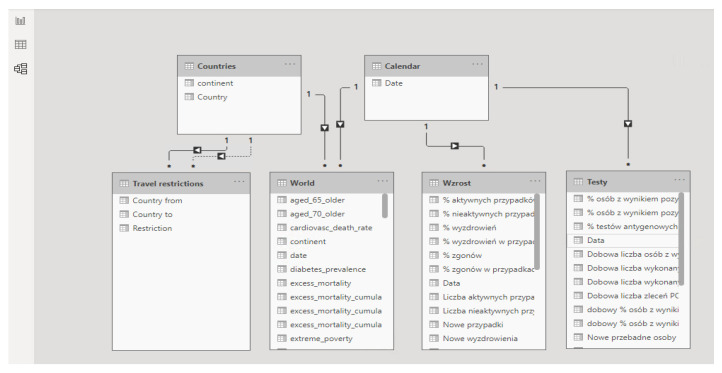
Relationship model of tables in Power BI in the presented example. Source: Authors own elaboration.

These two tables were added as connectors, so that other data sources could be joined to them. After all tables were prepared and properly transformed, it was then possible to set the necessary relations in the relationship model. This relationship model of tables in Power BI in the presented in Figure 5.

With properly set relationships, it is possible to use various tables in one visual and filter many visuals that use multiple tables using only the joining tables (Countries and Calendar).

Having prepared the data and relationships, it was then possible to prepare the visualizations. Power BI allows switching to mobile layout, which enables control over how the dashboard is presented on mobile devices. However, it is first necessary to have the prepared visuals in normal view and then to drag and drop them into the mobile layout.

**Figure 6:**
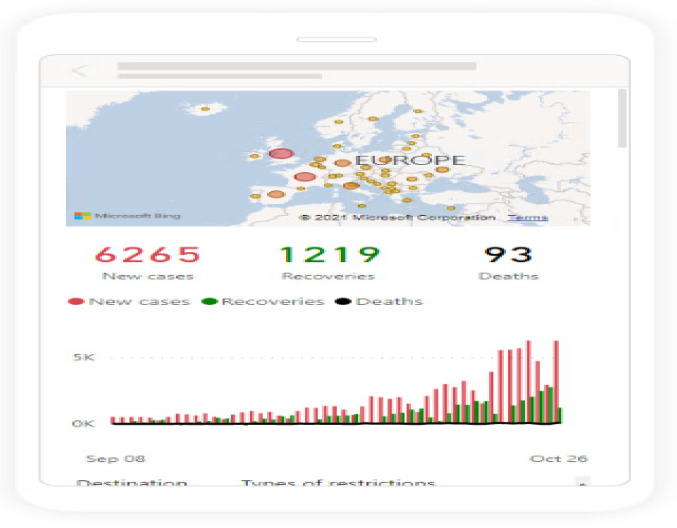
Example visualization in mobile layout prepared in Power BI. Source: Authors own elaboration.

In Figure 6, the example visualization in mobile layout prepared in Power BI is shown.

### 3.3. Application Prototype

The proposed application does not require the user to create an account or log in. It is more of an informational application that can be used to search for a travel destination during the pandemic. The prepared prototype includes:

- Start screen with logo created by the authors;
- Home page of the application;
- Useful information section;
- Statistics section with dashboard prepared in Power BI;
- Search section, where the user can browse for the guidelines on travel restrictions to the chosen destination, with a dashboard prepared in Power BI.

**Figure 7:**
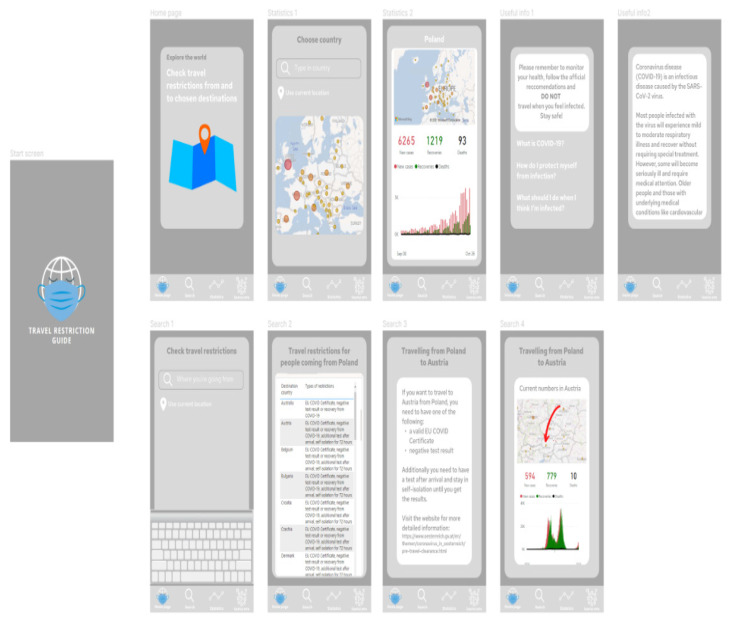
Project of the application screens—general view, showing all screens prepared in the prototype, starting from the home screen, choosing the location, Power BI report embedded in the application, additional information and statistics regarding COVID-19 in the chosen location, and table with travel restrictions. Source: Authors own elaboration.

**Figure 8:**
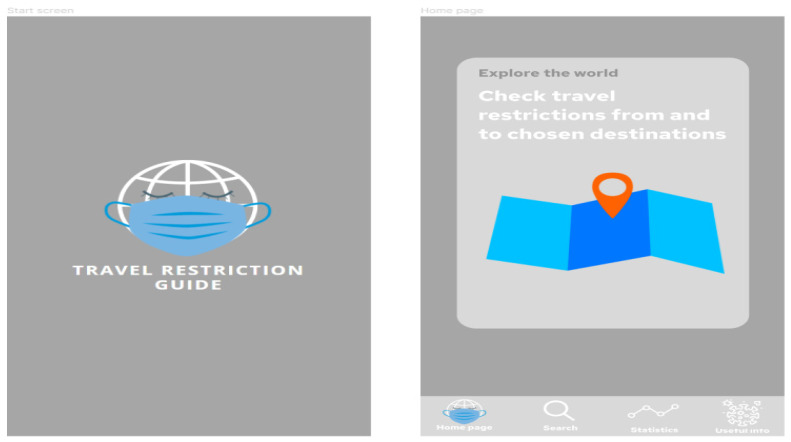
Start screen and home page of the application. These are the first screens that the user sees after launching the application. Source: Authors own elaboration.

**Figure 9:**
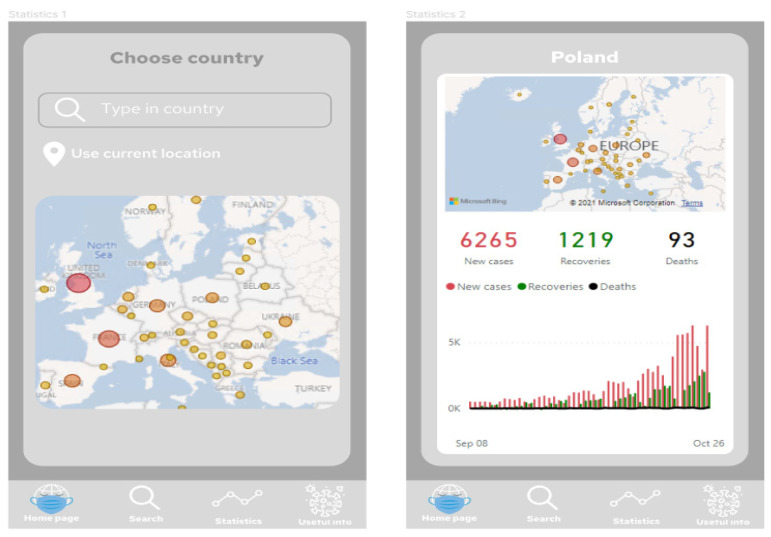
The first and second screen with the statistics in the application—choosing location and Power BI report embedded in the application. Source: Authors own elaboration.

**Figure 10:**
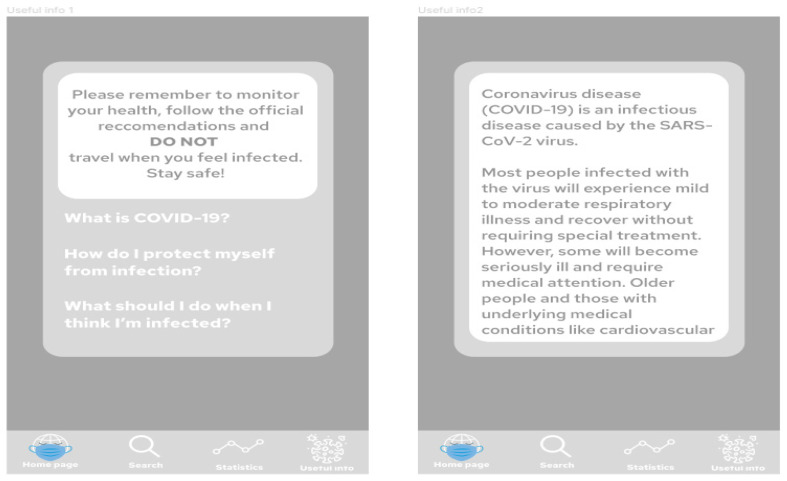
The first and second screen of the information section in the application, that can be chosen from the home screen, and states general information about COVID-19. Source: Authors own elaboration.

**Figure 11:**
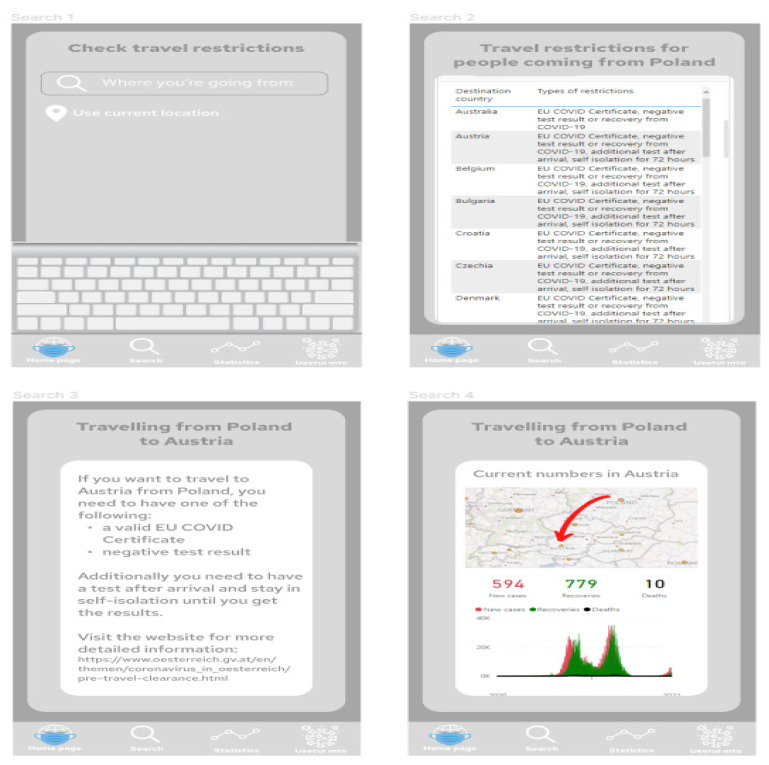
Four screens for searching travel destination and travel restriction details—description of the current restrictions to the chosen country and statistics about COVID-19 at the travel destination. Source: Authors own elaboration.

**Figure 12:**
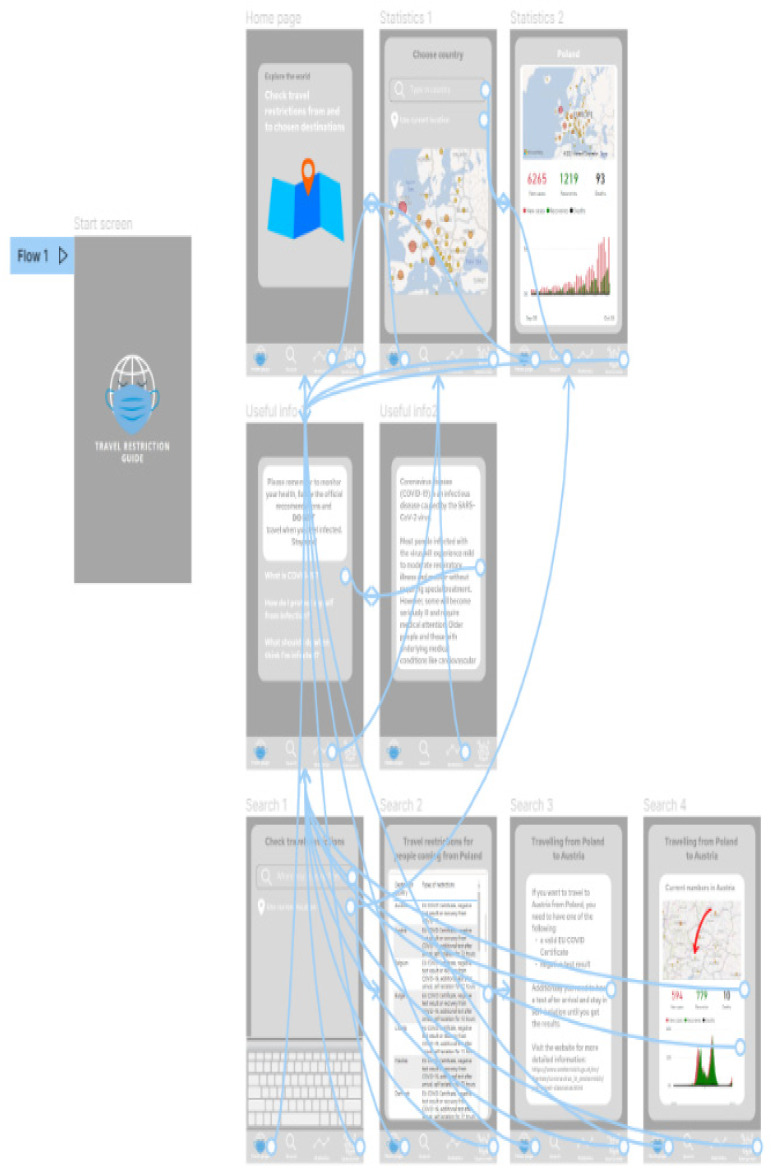
Project with transition arrows showing how the user can move between screens using particular buttons in the application. Source: Authors own elaboration.

Figure 7 presents project of the application screens—general view, showing all screens prepared in the prototype, starting from the home screen, choosing the location, Power BI report embedded in the application, additional information and statistics regarding COVID-19 in the chosen location, and table with travel restrictions. Figure 8 shows start screen and home page of the application. These are the first screens that the user sees after launching the application. Figure 9 presents the first and second screen with the statistics in the application—choosing location and Power BI report embedded in the application. It can be seen in Figure 10, the first and second screen of the information section in the application, that can be chosen from the home screen, and states general information about COVID-19. Figure 11 has shown four screens for searching travel destination and travel restriction details—description of the current restrictions to the chosen country and statistics about COVID-19 at the travel destination. Figure 12 has presented project with transition arrows showing how the user can move between screens using particular buttons in the application.

## 4. Discussion

The presented prototype of a mobile application for monitoring the global epidemiological situation, along with visualizations of COVID-19 data, is a basis for creating a real solution that could be used commercially to help people find simple, structured information about current travel restrictions and statistics. It is currently difficult to search for a single, ordered source of information about the travel restrictions around the world. The proposed dataset, as well as already existing and publicly available ones [52,53,54], can be further extended to include more detailed information, such as R ratio or predictions of the pandemic’s future development.

## 5. Conclusions

This paper focused on presenting the data visualization process and how to use it in a practical way, using the authors’ example of a mobile application that supports browsing for current travel restrictions. The proposed application itself is designed to help users find actual information about the restrictions based on official government publications and see the current numbers related to the COVID-19 pandemic.

A survey was conducted to examine people’s insights and feelings regarding the global epidemiological situation. Based on the survey results, it can be stated that most of the respondents believed that the travel restrictions will remain in some form for a long time. People typically search for information about these restrictions using sources such as news websites, but also obtain information from their friends and family. Individuals rarely use mobile applications related to COVID-19.

Following this, the authors went on to analyze the existing COVID-19-related tools and mobile applications in terms of their usability, popularity, and available functionalities. The authors distinguished the common features of the mobile applications and assigned grades for each one, compared the results, and found that ProteGO Safe [15] appeared to be the most appropriate application for users’ needs.

The steps of the data visualization process were presented, and examples were provided in relation to the proposed mobile application for monitoring the global epidemiological situation. Data visualization allows important information to be represented in ways that make it easier to understand. In order for data visualization to be prepared well, it is necessary to go through the process of defining the issue of analysis, finding or creating a data source, and then accurately transforming the data. This paper presented the possible ways to collect information about the travel restrictions in European countries. Following this, the types of data visualization were presented and described, including textual, tabular, and graphical data presentation. After this, the data visualization tools were introduced, including Microsoft Excel, Tableau, and Microsoft Power BI. The latter was used to present an example data visualization, where the mentioned data sources where utilized. The analyst needs to determine which of the many data visualization tools best fits their needs. The last step was to introduce the idea of prototyping, including its types and tools that help UX/UI designers to prepare such models. Again, an example of a mobile application for monitoring the global epidemiological situation was used as a base to prepare a sample prototype using Figma [14]. The last section described how important it is to define users’ requirements properly in the application development process, and emphasized the role of prototyping.

Based on their own idea of a mobile application for monitoring the epidemiological situation, the already existing solutions, research, and their own study, the authors presented a potential way of combining data visualization and mobile application prototyping that can be developed further. It could be a base for developers to build a real-life mobile application ready for use by end users to support them in the difficult times of the pandemic.

## Data Availability

Not applicable.
